# Assessment of Extramammary Paget Disease by Two-Photon Microscopy

**DOI:** 10.3389/fmed.2022.839786

**Published:** 2022-02-25

**Authors:** Radu Hristu, Lucian G. Eftimie, Stefan G. Stanciu, Remus R. Glogojeanu, Pavel Gheorghita, George A. Stanciu

**Affiliations:** ^1^Center for Microscopy-Microanalysis and Information Processing, University Politehnica of Bucharest, Bucharest, Romania; ^2^Pathology Department, Central University Emergency Military Hospital, Bucharest, Romania; ^3^Department of Special Motricity and Medical Recovery, The National University of Physical Education and Sports, Bucharest, Romania; ^4^Faculty of Energetics, University Politehnica of Bucharest, Bucharest, Romania

**Keywords:** second harmonic generation, two-photon excited fluorescence, extramammary Paget disease, immunohistochemistry, hematoxylin and eosin

## Abstract

Two-photon microscopy techniques are non-linear optical imaging methods which are gaining momentum in the investigation of fixed tissue sections, fresh tissue or even for *in vivo* experiments. Two-photon excited fluorescence and second harmonic generation are two non-linear optical contrast mechanisms which can be simultaneously used for offering complementary information on the tissue architecture. While the former can originate from endogenous autofluorescence sources (e.g., NADH, FAD, elastin, keratin, lipofuscins, or melanin), or exogenous eosin, the latter is generated in fibrillar structures within living organisms (e.g., collagen and myosin). Here we test the ability of both these contrast mechanisms to highlight features of the extramammary Paget disease on fixed tissue sections prepared for standard histological examination using immunohistochemical markers and hematoxylin and eosin staining. We also demonstrate the label-free abilities of both imaging techniques to highlight histological features on unstained fixed tissue sections. The study demonstrated that two-photon microscopy can detect specific cellular features of the extramammary Paget disease in good correlation with histopathological results.

## Introduction

Extensive efforts have been deployed over the past couple of decades for advancing the state-of-the-art in clinical diagnosis of malignant dermatological conditions (e.g., non-melanoma skin cancers). However, conventional histopathology assays that require small tissue fragments to be excised, fixed, stained, and subsequently studied by bright-field microscopy (BM) remains the gold standard for the diagnosis of such pathologies. It cannot be neglected though that such procedures exhibit important limitations, such as sampling error, slow speed, high costs, or interpretative variability (1), to name just a few. Such limitations result in a great need for alternative diagnosis methods that are faster and less invasive (2), while retaining the advantages of conventional histopathology, mainly those related to facile image interpretation. These would consistently improve the diagnosis workflows, reduce associated costs, and finally, and most importantly, result in increased survival rates of the patients.

Among the most promising solutions that have been proposed to date for the non-invasive assessment of malignant epithelial pathologies are magnetic resonance imaging (3), optical coherence tomography (4), and more recently, reflectance confocal microscopy (5) and non-linear optical (NLO) microscopy techniques (6). These latter exploit non-linear light-matter interactions (7) (e.g., two- or three-photon absorption, second or third harmonic generation) to achieve, in a label-free manner, high-contrast and easy to interpret images of tissues. Two prominent NLO techniques are two-photon excited fluorescence (TPEF) microscopy and second harmonic generation (SHG) microscopy which provide complementary information that has been demonstrated to date to be highly valuable for tissue state assessment. TPEF involves the simultaneous absorption of two photons whose combined energy is sufficient to induce a transition to an excited state (8) and the subsequent emission of a single photon with a slightly smaller energy than the total energy of the two excitation photons due to non-radiative processes (e.g., vibrational relaxation). In the case of SHG (9), two photons with the same energy interact with a non-linear material and are effectively combined via virtual energy states to generate a new photon with twice the energy of the initial photons. Most TPEF and SHG biomedical applications exploit endogenous properties of tissues (10), but applications that involve labeling with fluorescence dyes (11) and harmonophores (12) have also been reported. Among the most discussed TPEF sources of endogenous contrast are metabolic substrates (e.g., NADH and FAD), structural proteins (e.g., elastin and keratin), lipofuscins, and melanin. Hence, cells are perfect sources of two-photon excited autofluorescence. On the other hand, SHG microscopy has been successfully used for imaging a series of fibrillar structures within living organisms (e.g., collagen, myosin) with the limitation that only non-centrosymmetric molecules provide contrast. Collagen, the most abundant protein in the human body, is very effective in generating SHG signals (13), and most SHG bioimaging applications have been devoted to assessing structural changes of the extracellular matrix, a highly collagenous structure whose architecture can provide important information on the onset and progression of various diseases (14). The abundance of collagen in various organs motivated a wide palette of SHG applications, successfully addressing different tissue types ranging from zebrafish embryos (15) to porcine bone samples (16), thyroid (17), skin tissue (18, 19), pancreas (20), lung (21), and many others. TPEF and SHG are highly complementary, and many past works report their joint use to resolve subtle aspects of tissues and to provide images that recapitulate the most important histological features that allow histopathologists to assign an accurate diagnosis. Throughout this article we refer to the joint use of TPEF and SHG as two-photon microscopy (TPM).

TPM has enabled applications ranging from basic research to clinical practice investigations (22, 23). The versatility of the NLO techniques is intimately intertwined with their ability to probe fixed, *ex-vivo* and *in-vivo* tissues. While at first sight, the use of NLO techniques for imaging fixed tissues may seem to be biased by their overlap with BM of labeled tissues, which is obviously much more affordable and accessible, at closer look we find that NLO techniques not only provide similar information with that available with BM but can add additional insights that complement traditional histological features (24, 25). NLO microscopy images collected on unlabeled tissues (*in-vivo, ex-vivo*, or fixed) are known to recapitulate the most important features found in conventional histopathology. Nevertheless, it cannot be neglected that the aspect of such features is significantly different compared to that available in BM images of stained tissues, using either hematoxylin and eosin (H&E) or immunohistochemistry (IHC) staining. Given that histopathologists are not specifically trained to interpret such NLO images, a series of problems arise, with their most important consequences being the risk of false negatives or false positives. To address this issue, a consistent body of work has been devoted to virtual staining, that is assigning to NLO images the aspect of conventional histopathology images via either advanced color re-mapping schemes or modern artificial intelligence methods (26). Although such efforts are highly valuable and play an important role in facilitating NLO data interpretation, it should be noted that a universal image processing or artificial intelligence method capable to generalize and accurately address all tissue types is still missing. NLO images acquired on widely available H&E and IHC-stained tissue slides might facilitate the interpretation of NLO images collected on unlabeled tissues.

In this work, we focus on TPM imaging of the extramammary Paget disease (EMPD) which is a rare intraepithelial adenocarcinoma with clinical and histologic characteristics that mimic a range of inflammatory and infectious skin conditions. EMPD is more common in the anogenital region and other areas that are rich in apocrine glands, such as the axillae and the external ear. Clinically it presents as a slow-growing unilateral erythematous plaque, with well-defined irregular borders that frequently causes intense pruritus, or a burning sensation sometimes accompanied by hyper- or hypopigmented patches. Although it has controversial origins (27), EMPD is histologically characterized by a prominent growth of large cells. In the case of primary EMPD these cells may originate either in the ducts of the apocrine glands or in uncertain pluripotential keratinocytic cells (Toker cells). On the other hand, secondary EMPD represents an intraepithelial spread of a hidden primary adenocarcinoma. EMPD is typically multicentric and it is therefore necessary to perform several biopsies to map the contours of the lesion. The standard treatment is the surgical excision of the tumor by performing either wide excision or Mohs micrographic surgery (28). However, margin control is challenging due to its non-specific clinical appearance and subclinical spread. Furthermore, non-surgical treatment responses are variable and difficult to monitor, and recurrence rates are high.

Various imaging approaches including TPM were previously used to characterize Paget cells and their spreading pattern. For example, the subclinical extension was assessed by TPEF using whole-mount immunostaining with cytokeratin 7 to label Paget cells (29), while artificial intelligence approaches applied on NLO images were used for margin assessment of EMPD (30). With this study, we provide additional insights on TPM's capability to provide valuable support on NLO data interpretation and to visualize the EMPD nests and their cellular morphology. The TPM results obtained on unstained tissues fragments are compared with those available by BM on tissue sections as prepared for standard histology with H&E and IHC staining. We hypothesize that our results obtained on unstained fixed tissue sections can be straightforward extrapolated to *ex-vivo* or *in-vivo* settings, considering the more intense TPM signals typically exhibited by freshly excised or living tissues. Moreover, an additional use for NLO imaging of fixed tissues labeled for conventional histopathology is proposed.

## Materials and Methods

Thin serial sections (4–7 μm) were cut from the formalin-fixed, paraffin-embedded tissue block, mounted on glass slides, and stained with H&E or following the standard IHC protocols. IHC staining markers which are usually used for the differential diagnosis of EMPD were used in this study. Paget cells are positive (31) when stained for Cytokeratin 7 (CK7**)**, epithelial membrane antigen (EMA), which is usually expressed in Paget cells which contain sialylated intracellular EMA in both extramammary and mammary Paget's disease (32), and gross cystic disease fluid protein-15 (GCDFP-15) (33). We have also used IHC staining for the human epidermal growth factor receptor 2 (HER2/neu) (34), human melanoma black 45 (HMB45) and melanoma antigen (MelanA). The use for scientific research purposes of patient samples was approved by the Ethics Committee from Carol Davila University Central Emergency Military Hospital, Bucharest, Romania, and written informed consent was obtained from the patients. All methods were performed according to relevant guidelines and regulations and in accordance with the Declaration of Helsinki.

All slides were imaged with a Zeiss Axio Imager.M2 bright-field microscope (Carl Zeiss Microscopy GmbH, Germany) for determining the regions of interest (ROIs) which were further imaged by TPM.

Combined SHG and TPEF images (to which we refer in the following as TPM images) were acquired using a Leica TCS-SP confocal laser scanning microscope modified for NLO imaging (35). The excitation source was a Ti:Sapphire laser (Chameleon Ultra II, Coherent) tuned at 870 nm, with ~140 fs pulses and a repetition rate of 80 MHz. The input excitation laser beam was circularly polarized by a combination between an achromatic quarter-wave plate (AQWP05M-980, Thorlabs) and an achromatic half-wave plate (AHWP05M-980, Thorlabs) placed in the laser beam path before the microscope input port. A 40 × magnification and 0.75 numerical aperture (NA) objective was used for focusing the laser beam on the sample and for collecting the TPEF signals. The SHG signals were collected in the forward direction using a 0.9 NA condenser lens. A shortpass filter (FF01-750/SP-25, Semrock, Rochester, New York) combined with a bandpass filter (FB430-10, Thorlabs, Newton, New Jersey, United States) placed before the detector were used for filtering the SHG signals. The TPM images are presented as composite images with the TPEF signal on the red channel and the SHG signal on the blue channel. Purple areas represent co-localized TPEF and SHG signals. In the cases where no collagen was present in the imaged field-of-view, no SHG signal was detected, hence no signal on the blue channel in the composite TPM image.

## Results

Bright-field microscopy and TPM images were acquired on different ROIs on (i) H&E-stained sections, (ii) IHC-stained sections using six different markers, and (iii) unstained tissue sections, respectively. Although the stained and unstained tissue sections have been extracted from the same histological block, and are comprised of the same tissular structures, small morphological variation occur from section to section. Representative ROIs for each studied case are presented in the following and features visible in the TPM images are discussed. Bright-field microscopy and TPM images were acquired sequentially and registered in ImageJ using the “Align image by line ROI” plugin.

**Figure 1:**
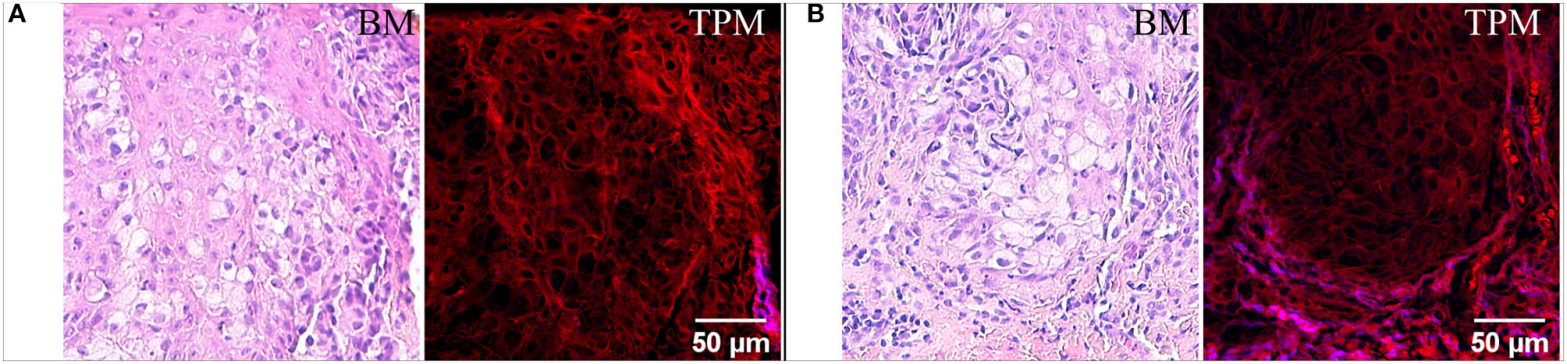
Bright-field microscopy (BM) and TPM images of vulvar tissue acquired on H&E-stained sections. **(A)** Imaged area closer to the epidermis surface. **(B)** Imaged area at the dermoepidermal junction.

**Figure 2:**
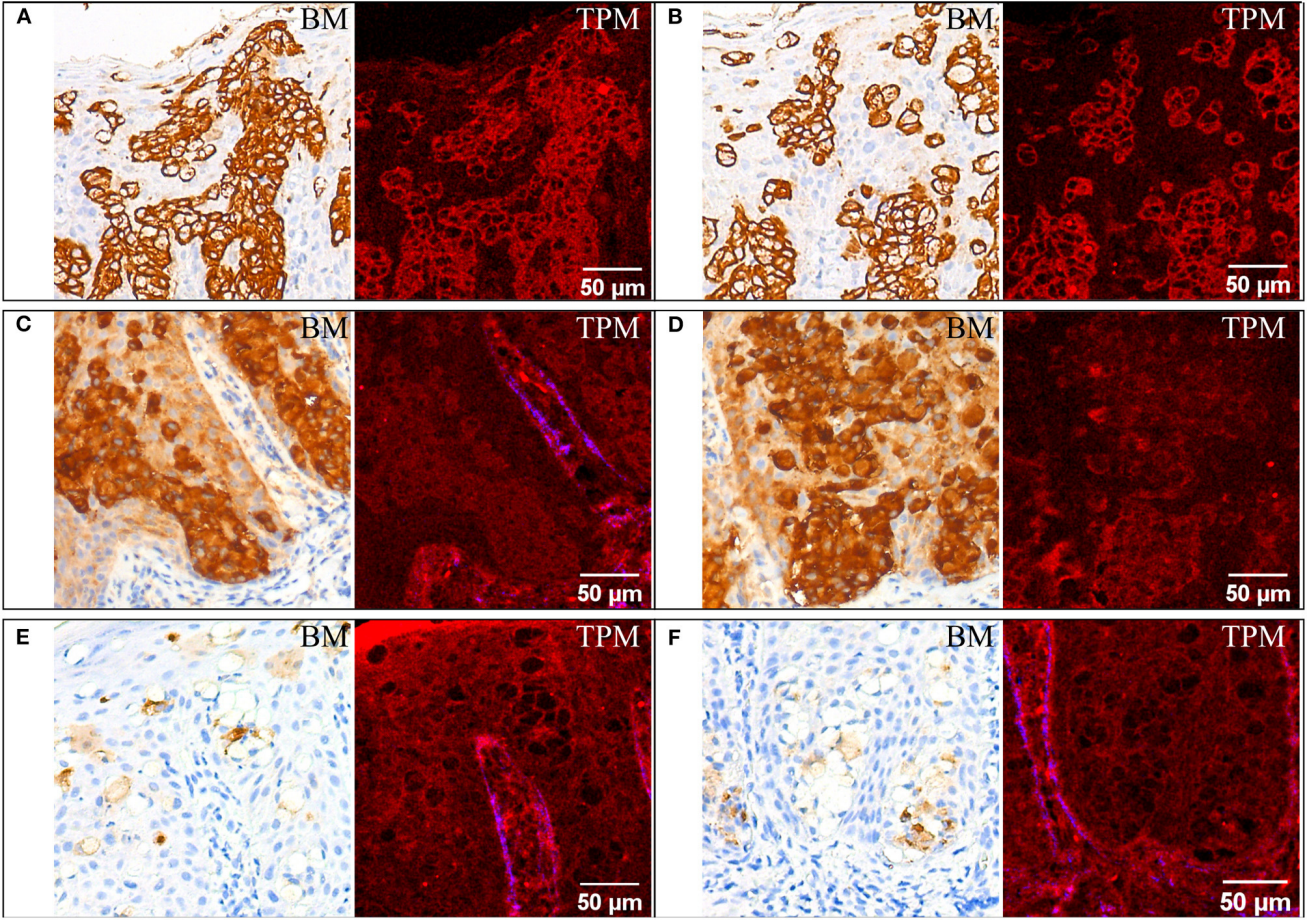
Immunohistochemical staining for CK7 **(A,B)**, EMA **(C,D)**, and GCDF-P15 **(E,F)**. For each staining two ROIs are provided, each with bright-field microscopy (BM) and corresponding TPM images. For **(A,B,D)** the SHG signal on the blue channel in the TPM image is almost absent since no collagen is present in the ROIs.

Images acquired with bright-field microscopy on H&E-stained tissue sections (Figure 1), highlight several polygonal or round-oval shaped large glandular cells with pale abundant cytoplasm which may contain small vacuoles and a big nucleus often pushed to a narrow peripheral rim which infiltrate in the epidermis. These neoplastic cells are spread throughout the epidermis (Figure 1A), can be arranged in nests (Figure 1B) or in a trabecular pattern (Figure 2A), usually situated above the basal layer of the epidermis. In the TPM images acquired on H&E-stained sections (Figure 1) the TPEF signal originates in this case from the eosin-stained structures. Usually, eosin, which is a negatively charged acidic dye, non-specifically stains acidophilic structures, such as cytoplasm proteins, borders of the cell membrane, red blood cells and extracellular structures (including collagen), resulting in features visible in bright-field microscopy in varying degrees of pink. In the case of the TPM images depicted in Figure 1, we can observe that the same polygonal or round/oval cells are visible as in the optical microscopy images, however without clearly visible nuclei. The aspect of Paget cells in the acquired TPM images, dark structures with a very dim fluorescence, resembles to their aspect in reflectance confocal microscopy (36) where they were observed as hyporeflective structures. The Paget cells above the basal layer of the epidermis (Figure 1A), which are visible in both TPM, and bright-field microscopy images are most probably derived from it.

Paget cells show strong complete membrane positive labeling (represented in walnut or brown in bright-field microscopy images in Figures 2A,B) when using an antibody against cytokeratin 7 (CK7), which is specifically expressed in Paget cells of EMPD as well as in normal structures of eccrine and apocrine ducts/glands. Paget cells can also be easily highlighted using TPM images as brightly red colored cellular membranes in Figures 2A,B.

Paget cells are intensely positive for EMA (Figures 2C,D). They are also clearly visible in the TPM images obtained on tissue sections stained for EMA, located close to the dermoepidermal junction in the epidermis. Compared to the case of tissue sections stained for CK7, where Paget cells have bright cellular membranes, in this case the contrast is undifferentiated for cellular features, and it is lower than before, even if the Paget cells are positive for both CK7 and EMA.

Paget cells are moderately positive for GCDFP-15 (Figures 2E,F). Similar with the case of H&E staining (Figure 1), the Paget cells appear as optically free in both optical microscopy and TPM images and can be differentiated from the rest of the squamous cells of the epidermis which have indistinguishable cellular limits in the TPM image.

**Figure 3:**
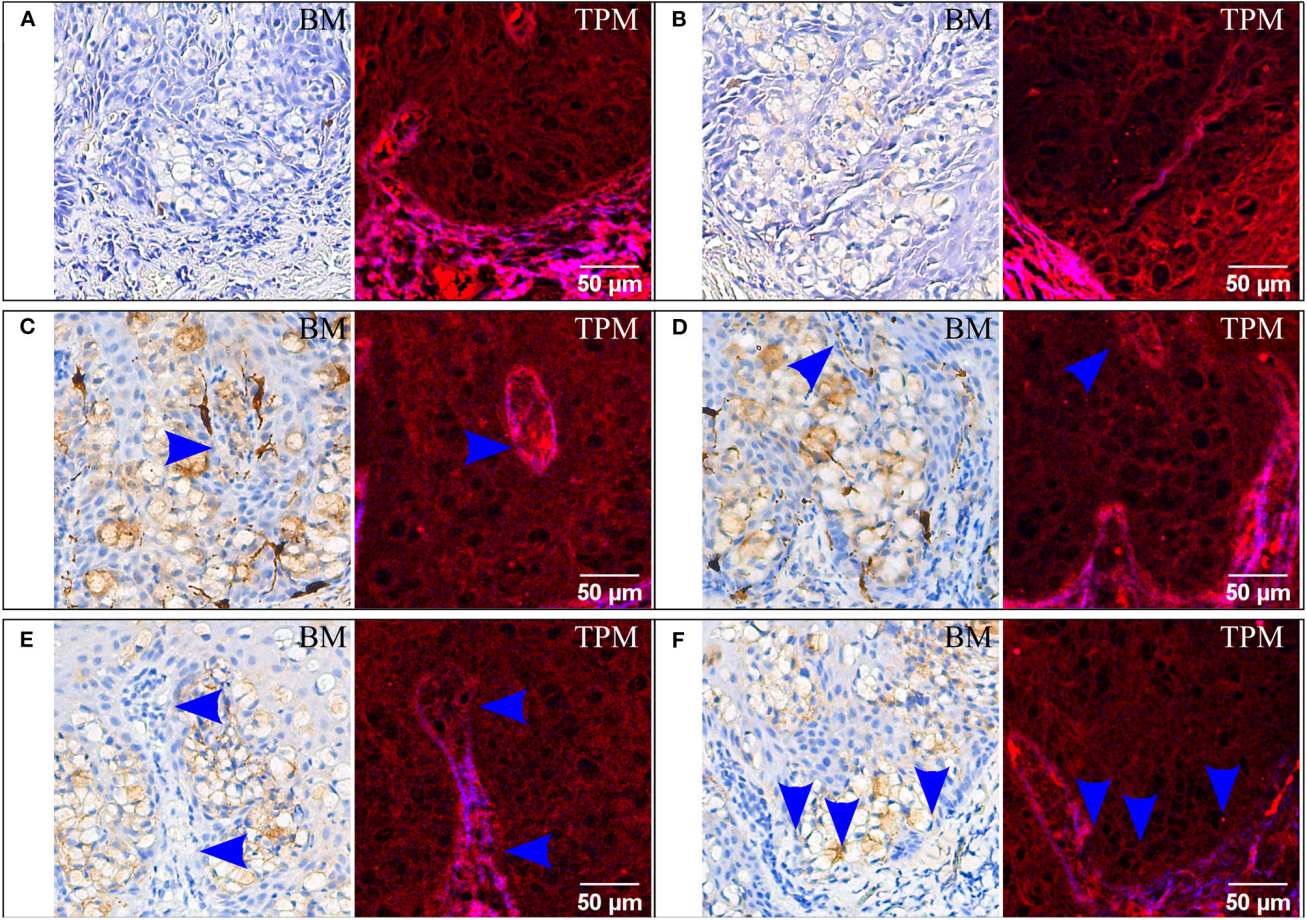
Immunohistochemical staining for HMB45 **(A,B)**, MelanA **(C,D)**, and HER2/neu **(E,F)**. For each staining two ROIs are provided, each with bright-field microscopy (BM) and corresponding TPM images. In **(C,D)** arrows indicate false islands of neoplastic cells which are transversally sectioned intraepidermal papillae from the dermis. In **(E,F)** possible invasion sites are marked with arrows; the brightly colored red and blue dermis in the TPM images is invaded by a group of cells with dim cytoplasm.

Other histological aspects can also be revealed by analyzing the TPM images in Figures 2E,F. The larger lips (labia majora) of the vulva develop from labioscrotal folds, which have an ectodermal origin. This explains why they are covered by skin and share many characteristics with the skin on different parts of the body. One of these common features relates to the layered structure of the skin on the larger lips or from any other body parts which has a superficial layer, the epidermis (a keratinized stratified squamous epithelium). Figure 2E shows the surface keratin layer (bright red in the upper left corner of the TPM image) and the prominence of the dermoepidermal junction by emphasizing the papillary dermis, the areas known as dermal papillae which are colored in blue (SHG signal from collagen) and shows the invagination of the epidermis (also clearly visible in Figure 2C). This delimitation is unnoticeable on bright-field images acquired on the corresponding section stained for GCDFP-15 (Figures 2E,F). It is necessary that we observe the dermoepidermal junction to discover possible focal invasions of the tumor cells in the papillary dermis. The next layer, the dermis, which is thicker than the epidermis, is composed of the papillary and the reticular dermis. These two are less well-defined in the vulva than they are in other skin regions. The dermis of the labia majora contains collagen which is visible in the lower part of Figure 3A in blue—SHG signal in the TPM image. In general, it may also contain elastic fibers, blood vessels, nerves but also hair follicles, apocrine and eccrine sudoriparous glands in the outer regions and sebaceous glands, which are found both in the outer and inner regions. The fragment of vulvar tissue containing Paget cells chosen for the TPM examination does not exhibit either hair follicles in the dermis or apocrine and eccrine sudoriparous or sebaceous glands, to emphasize the collagen in the papillary dermis without any adnexal structures.

HMB45 is a common marker used to confirm melanoma and was employed here for the differential diagnosis (31) of EMPD. In the case of tissue sections stained for HMB45 (Figures 3A,B), Paget cells are negative in the bright-field microscopy images, and they are detectable in the TPM images.

For the same differential diagnosis algorithm, one uses MelanA, which should be positive for superficial spreading melanoma and negative for both primary and secondary EMPD (31). In our case the result is false positive (bright-field images in Figures 3C,D). It is thus noteworthy to observe that Paget cells are visible in TPM images (Figures 3C,D), without the influence of the IHC marker used or the result obtained by IHC staining.

Another interesting aspect which can be better visualized in TPM images are the false islands of neoplastic cells which actually are transversally sectioned intraepidermal papillae from the dermis (marked with an arrow in Figures 3C,D). These are difficult to detect in the IHC-stained sections and imaged by bright-field microscopy (Figures 3C,D).

Similar detectable Paget cells groups on the sections stained for HER2/neu can also be found in the TPM images (Figures 3E,F). Moreover, a possible invasion site can be noticed in the papillary dermis and is only visible in the TPM image. At the tip of the invagination in Figure 3E (blue arrows) the dermoepidermal junction visible in SHG contrast in the TPM images is no longer intact and it is interrupted by large cells with dim cytoplasm which were previously identified as Paget cells. The examination of the bright-field microscopy images alone, where the tumor cell proliferation is intraepidermal and no invasion site is evident, would result in a diagnosis of non-invasive or “*in situ*” EMPD. It is thus crucial to correctly identify invasion sites since the prognosis is much worse in cases where the Paget cells have invaded the dermis from the epidermis (37).

**Figure 4:**
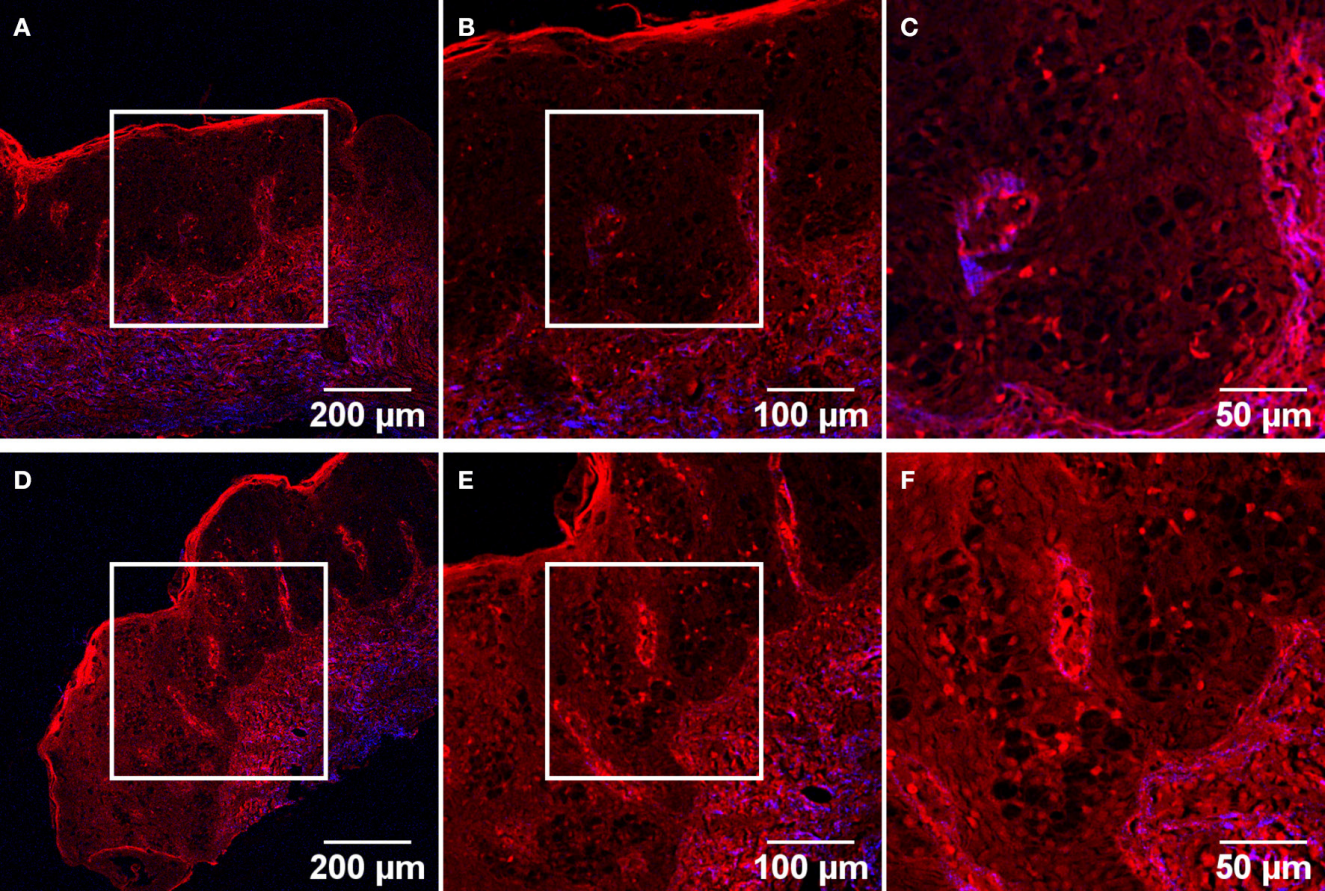
TPM images of two different ROIs acquired on an unstained tissue section. Objectives with 10X **(A,D)**, 20X **(B,E)**, and 40X **(C,F)** magnification were used, for each magnification the image being acquired in the center of the previous field-of-view (corresponding squares).

The most detailed images in term of tissular features were obtained on unstained tissue sections, hence on samples for which images without an identifiable morphology would be obtained by conventional bright-field microscopy. The same histological features as described before can also be detected using label-free TPM imaging. Starting from the wide field-of-view (FOV) images (Figures 4A,B,D,E) the skin layers can easily be observed: the epidermis, with a brighter upper keratin layer, and underneath it the dermis in a brighter red color with the papillary structure. There is a thin demarcation line between the two: the dermoepidermal junction. The papillary dermis composed predominantly of type III collagen mixed with type I collagen and fine elastic fibers has a low SHG signal (blue channel in TPM images) since type III collagen is a poor SH generator, hence the intense red color corresponding to two-photon excited autofluorescence. The deeper dermis layer, the reticular dermis is composed of well-organized type I collagen, arranged parallel with the skin surface. The reticular dermis can be easily separated from the papillary dermis since it is appearing in a blue/purple color because of the SHG signal and co-localization between TPEF and SHG signals (purple in TPM images) originating from collagen type I which predominates here. The main feature of EMPD which can be identified by TPM in the higher magnification images (Figures 4C,F) is the presence of groups of large polygonal shaped cells with dim cytoplasm in the epidermis. These cells have been previously identified as Paget cells by comparing bright-field images acquired on H&E or IHC-stained tissue sections with TPM images obtained at matching locations (see Figures 1–3).

## Discussions

Two-photon microscopy (TPM) was used to assess tissue sections of vulvar origin for features characteristic to EMPD, including individual Paget cells and tumor nests at the dermoepidermal junction. Two contrast mechanisms were used to obtain images on H&E and IHC stained as well as unstained tissue sections. Two-photon excited fluorescence (TPEF) was responsible for optical signals originating from endogenous autofluorescence sources in the unstained and IHC-stained tissues sections. 3,3'-Diaminobenzidine (DAB) was the chromogen used for visualization of horseradish peroxidase activity in the IHC assays. Because the product of the enzymatically catalyzed oxidation of DAB by hydrogen peroxide is brown and non-fluorescent (38) and the tissue was counterstained with hematoxylin which is also non-fluorescent, the only source for fluorescence is the endogenous one in IHC stained tissue. It is noteworthy to mention that due to the IHC protocol which involved blocking non-specific binding by using non-ionic detergents and hydrogen peroxide and thermal treatment for antigen retrieval, tissue autofluorescence becomes dimmer. For H&E-stained tissue sections, eosin which binds non-specifically to proteins within the cytoplasm, borders of the cell membrane, red blood cells and extracellular structures (including collagen) was responsible for the TPEF signal. The second TPM contrast mechanism which we used, second harmonic generation (SHG) originates from collagen fibers within the sample, regardless of the tissue being stained or not.

We were able to identify Paget cells in the TPM images of the H&E-stained sections, as well as in the IHC-stained sections, regardless of these cells were positive or negative for the corresponding markers. On the other hand, Paget cells were also observed in label-free TPM imaging (Figure 4), without performing any IHC assays (which are quite expensive, delay the diagnosis, and present the risk of obtaining a false positive or false negative result for these types of cells). Paget cells were identified as large cells with pale cytoplasm in the epidermis, near the dermoepidermal junction or in the papillary dermis. Because EMPD can mimic a lot of benign or malignant diseases, a correct differential diagnosis is crucial for the treatment and prognosis of the patient. So far, by using TPM images we have not obtained a differential diagnosis algorithm of these types of cells. It is also imperative to differentiate primary EMPD from the secondary type, while in the latter case the identification of the primary site of the tumor being vital for the patient. While these aspects are to be resolved in future experiments, we were still able to assess an invasion site which was not detected by analyzing only the bright-field microscopy images (Figures 3E,F).

*In-vivo* applications (39, 40) are at the forefront of non-linear optical (NLO) microscopy (which includes TPM) as they enable taking clinical decisions in real-time, overcoming the requirement of excisional biopsies and intrinsic connected disadvantages such as sampling error and invasivity. Additionally, *in-vivo* NLO imaging provides superb possibilities for the live monitoring of tissue modifications (41), and diagnosis follow-up (42). Imaging *ex-vivo* tissues with NLO is also very important, as the immediate analysis of freshly excised tissues (41), next to the patient, speeds up decision making, improving the overall efficiency of the medical act. NLO applications implemented on fixed tissues have an important role in facilitating the deeper penetration of NLO techniques in clinical applications, paving the way to next-gen medicine: fixed tissues (both stained and unstained) are abundant in histopathology labs worldwide, representing a valuable resource for designing at proof-of-concept level various NLO methods for diseases diagnosis, that can later be transferred to *ex-vivo* or *in-vivo* settings, at an optimal moment when they are already available at high technology readiness level. As well-known, *ex-vivo* and *in-vivo* experiments carry significant ethical implications, thus it is essential that NLO diagnosis methods are first developed and validated in settings that do not pose such concerns before they are finally transferred to clinical applications. In this regard, one might foresee three major technical limitations in performing such a study: the limited FOV, 250 × 250 μm^2^ for a 40 × objective used in this study, the penetration depth (~200 μm) and the weak signals originating from the unstained tissue would probably be the main technical challenges. Scanning large areas of the lesions is important to avoid false-negative diagnoses because lesions are often non-uniform. Finding potential invasion sites is also restricted with a limited FOV. The FOV can be increased by acquisition of adjacent ROIs and using mosaicking algorithms to stitch individual images. High penetration depth is important for imaging tumor nests far from the dermoepidermal junction in the dermis, which is the case of invasion. One way to increase penetration depth is by using tissue clearing methods which have improved in the past years and are currently available also for *in vivo* applications (43). Optical clearing utilizes exogenous agents which reduce the light scattering in biological tissues by refractive index matching and enhance image contrast. Optical clearing has been shown to increase the capabilities of TPEF (44) and SHG collagen imaging (45). Because TPM uses weak endogenous fluorescence and SH signals in tissue, there is a need for high excitation laser power and/or increase of pixel dwell times which are usually not compatible with *in-vivo* imaging. We find important to mention that NLO signals collected on fixed tissues are dimmer compared to those collected on freshly excised or *in-vivo* tissues, hence extrapolating results obtained on fixed tissues to *ex-vivo* settings might be straightforward from a technical point of view. *In-vivo* experiments are more demanding in terms of signal intensity, exposure time and photodamage. Hence, to overcome this limitations, moxifloxacin has been previously reported (46) as a cell-labeling agent for TPM. Moxifloxacin has bright TPEF, offers good tissue penetration, and high intracellular concentration. In addition, moxifloxacin-based TPM imaging is up to 10 times faster than imaging based on endogenous fluorescence (11) with similar photon counts.

Our results obtained on unstained tissue sections demonstrate that TPM is a good candidate to provide rapid on-site pathological analysis by label-free imaging with subcellular resolution, eventually without the need of a skin biopsy or physical sectioning of the tissue. For example, in the case of EMPD, proper tissue sampling by TPM can determine if there is an associated underlying malignancy that requires surgical excision. If such a malignancy exists, it should be excised along with all clinically abnormal epithelium. The features of TPM can also be used in conjunction with Mohs micrographic surgery (47), which involves precise progressive surgical removal of tumoral skin tissue with minimal healthy tissue removal depending on tumoral stage. Since Mohs surgery requires micrographic tumor imaging between excision stages to provide surgical guidance and final confirmation *via* frozen section pathology of clear margins (48), TPM might be an imaging method worth considering in a two-fold perspective. In a first stage, for *ex vivo* use, the surgical margins are removed and TPM is used to confirm whether the tumor is still there. The second stage would be for *in vivo* application in a later implementation when TPM can help in the identification of the surgical margins in a perioperative setting. However, when used for detection in Mohs surgery, the TPM images would be difficult to interpret by the surgeons. In this work we propose an additional use for TPM imaging of fixed tissues stained for conventional histopathology. The problem that we address is data interpretation, which is still a consistent bottleneck that obstructs the further penetration of TPM imaging to clinical applications. Despite its advantages, TPM is not widespread due, in part, to the need for pathologists and surgeons to retrain to interpret TPM images. To address this issue, a consistent body of work has been devoted to virtual staining, which assigns NLO images the aspect of conventional histopathology images. Although such efforts are highly valuable and play an important role in facilitating NLO data interpretation, to date, a universal method capable to generalize and accurately address all tissue types is still missing. Thus, an alternative strategy could be to assemble public libraries of NLO images on various types of tissues, so that histopathologists worldwide can become accustomed to the aspect of NLO data and thus enhance their NLO interpretation skills. This route is also paved with a series of difficulties. For instance, access to freshly excised or even fixed unlabeled tissues, is difficult, as usually in the frame of the medical procedure tissue fragments of minimal size are extracted, to avoid patient scarring and trauma, and these are usually fixed and stained, to be used for diagnosis purposes in conventional histopathology assays. On the other hand, hospitals and research institutes worldwide harbor large collections of stained histology slide, which after diagnosis are usually kept for several years. We argue that using NLO images collected on this widely available category of tissue samples, can facilitate the interpretation of NLO images collected on unlabeled tissues, thus it is a solution worthy to explore. This proof-of-concept experiment demonstrates the utility of such assays for NLO data interpretation. A concerted effort aiming to assemble a public repository of NLO images collected on stained tissues could massively facilitate the interpretation of NLO data, and thus promote the further penetration of NLO imaging in clinical applications devoted to *ex-vivo* and *in-vivo* use. Moreover, current software applications which perform digital and 3D analyses on standard H&E images can be extended to TPM images (18). This approach can improve the accuracy of the EMPD diagnosis and possibly to other neoplasms (e.g., clear cell Bowen's disease and superficial spreading amelanotic melanoma).

## Conclusions

This observational pilot study demonstrated that two-photon microscopy can detect specific cellular features of the extramammary Paget disease in good correlation with histopathological images. Our study shows that two-photon microscopy (with the two contrast mechanisms two-photon excited fluorescence and second harmonic generation) on fixed tissue sections can resolve the cellular structure inside the Paget cells nests. This is important because the presence of Paget cell groups at the dermoepidermal junction and/or in the dermis is the main histopathologic criterion for EMPD diagnosis. Therefore, this feature can be potentially used for diagnosis and prognosis of EMPD with two-photon microscopy.

## Data Availability Statement

The raw data supporting the conclusions of this article will be made available by the authors, without undue reservation.

## Ethics Statement

The studies involving human participants were reviewed and approved by Ethics Committee from Carol Davila University Central Emergency Military Hospital, Bucharest, Romania. The patients/participants provided their written informed consent to participate in this study.

## Author Contributions

RH and LE collected and analyzed the data and drafted the manuscript. GS supervised the project and reviewed the manuscript. SS conceived of the study and helped to draft the manuscript. RG and PG analyzed the data. All authors read and approved the final manuscript.

## Funding

This work was supported by the Romanian Executive Agency for Higher Education, Research, Development and Innovation Funding (UEFISCDI) under the grant PN-III-P1-1.1-TE-2019-1756 (SHGThyPath). The use of the Ti:Sapphire laser (Chameleon Ultra II, Coherent) was possible due to European Regional Development Fund through Competitiveness Operational Program 2014–2020, Priority axis 1, Project No. P_36_611, MySMIS code 107066, Innovative Technologies for Materials Quality Assurance in Health, Energy and Environmental—Center for Innovative Manufacturing Solutions of Smart Biomaterials and Biomedical Surfaces—INOVABIOMED.

## Conflict of Interest

The authors declare that the research was conducted in the absence of any commercial or financial relationships that could be construed as a potential conflict of interest.

## Publisher's Note

All claims expressed in this article are solely those of the authors and do not necessarily represent those of their affiliated organizations, or those of the publisher, the editors and the reviewers. Any product that may be evaluated in this article, or claim that may be made by its manufacturer, is not guaranteed or endorsed by the publisher.
